# Controlling a VRE cluster on a surgical ward

**DOI:** 10.1186/2047-2994-4-S1-P200

**Published:** 2015-06-16

**Authors:** C Bandiera-Clerc, C Coccault-Duverger, F Bellissimo-Rodrigues, P Albrecht, G Renzi, F Ris, S Harbarth

**Affiliations:** 1 Infection Prevention and Control service, Geneva University Hospitals, Switzerland; 2 Visceral Surgery service, Geneva University Hospitals, Switzerland; 3 Hygiene and Property service, Geneva University Hospitals, Switzerland; 4 Bacteriology Laboratory, Geneva University Hospitals, Switzerland

## Introduction

Vancomycin-resistant *Enterococcus faecium* (VRE) has been previously described to be responsible for large nosocomial outbreaks, with a relevant clinical impact, in terms of morbidity and mortality, and health-economic burden.

## Objectives

To describe a VRE cluster and to evaluate the impact of the control measures implemented.

## Methods

This was a prospective outbreak investigation addressing the impact of a bundle strategy on a VRE cluster which took place in two adjacent surgical wards comprising 58 beds, University of Geneva Hospitals from September 1, 2014 to December 2, 2014.

The implemented bundle strategy consisted in: screening of all patients in the wards by rectal swabs, promoting hand hygiene compliance and patient education, implementation of contact precautions with private room allocation for all colonized patients, daily disinfection of environmental surfaces and medical devices with a 80% mono peroxyphthalate-based disinfectant.

## Results

The index case of the cluster was a patient with cognitive disability which freely circulated within the hospital wards and had been hospitalized for 3 weeks before a urine culture yield VRE, despite the absence of related symptoms. Following that discovery, 141 patients were screened by rectal swab, yielding 5 other VRE-colonized patients (attack rate = 4,25%) and, fortuitously, 10 patients colonized by Extended-Spectrum β-Lactamase (ESBL) producing-*Klebsiellapneumoniae*. The bundle strategy was implemented soon after the discovery of the index case, and remained on for 8 weeks, effectively controlling both clusters. The decision to stop it was based on the fact that no new cases of VRE or ESBL colonization were detected by rectal screening for a period of 4 weeks. After the intervention was discontinued, no additional cases were found in the following month (2 point prevalence performed).

Fortunately, no cases of VRE-related infections were detected, and only one patient developed peritonitis due to the ESBL-producing *K. pneumoniae*, and survived to it.

## Conclusion

A VRE-cluster among surgical patients was effectively controlled by the fast implementation of a bundle intervention consisting mainly of reinforced hand hygiene, environmental disinfection, screening and strict contact precautions.

## Disclosure of interest

None declared.

